# Divergent Antiviral Mechanisms of Two *Viperin* Homeologs in a Recurrent Polyploid Fish

**DOI:** 10.3389/fimmu.2021.702971

**Published:** 2021-08-31

**Authors:** Cheng-Yan Mou, Shun Li, Long-Feng Lu, Yang Wang, Peng Yu, Zhi Li, Jin-Feng Tong, Qi-Ya Zhang, Zhong-Wei Wang, Xiao-Juan Zhang, Guang-Xin Wang, Li Zhou, Jian-Fang Gui

**Affiliations:** ^1^State Key Laboratory of Freshwater Ecology and Biotechnology, Institute of Hydrobiology, The Innovative Academy of Seed Design, Chinese Academy of Sciences, Wuhan, China; ^2^College of Life Sciences, University of Chinese Academy of Sciences, Beijing, China; ^3^Hubei Hongshan Laboratory, Chinese Academy of Sciences, Wuhan, China

**Keywords:** *viperin*, polyploid, homeolog, herpesvirus, proteasomal degradation, autophagosome

## Abstract

Polyploidy and subsequent diploidization provide genomic opportunities for evolutionary innovations and adaptation. The researches on duplicated gene evolutionary fates in recurrent polyploids have seriously lagged behind that in paleopolyploids with diploidized genomes. Moreover, the antiviral mechanisms of Viperin remain largely unclear in fish. Here, we elaborate the distinct antiviral mechanisms of two *viperin* homeologs (*Cgviperin-A* and *Cgviperin-B*) in auto-allo-hexaploid gibel carp (*Carassius gibelio*). First, *Cgviperin-A* and *Cgviperin-B* showed differential and biased expression patterns in gibel carp adult tissues. Subsequently, using co-immunoprecipitation (Co-IP) screening analysis, both *Cg*Viperin-A and *Cg*Viperin-B were found to interact with crucian carp (*C. auratus*) herpesvirus (*Ca*HV) open reading frame 46 right (ORF46R) protein, a negative herpesvirus regulator of host interferon (IFN) production, and to promote the proteasomal degradation of ORF46R *via* decreasing K63-linked ubiquitination. Additionally, *Cg*Viperin-B also mediated ORF46R degradation through autophagosome pathway, which was absent in *Cg*Viperin-A. Moreover, we found that the N-terminal α-helix domain was necessary for the localization of *Cg*Viperin-A and *Cg*Viperin-B at the endoplasmic reticulum (ER), and the C-terminal domain of *Cg*Viperin-A and *Cg*Viperin-B was indispensable for the interaction with degradation of ORF46R. Therefore, the current findings clarify the divergent antiviral mechanisms of the duplicated *viperin* homeologs in a recurrent polyploid fish, which will shed light on the evolution of teleost duplicated genes.

## Introduction

Genome sequencing explosion has highlighted a profound impact of polyploidy and subsequent post-polyploid diploidization (PPD) on evolutionary innovations and adaptation. As the impact might lead to genomic diversity, variability, and complexity, polyploidy is generally considered to have far-reaching consequences in shaping species speciation, diversification, and ecological adaption (1–10). The evolutionary fates of duplicated genes are dynamic and complex and include pseudogenization or gene deletion, subfunctionalization, or neofunctionalization under relaxed purifying selection (3). In animals, the evolution of duplicated genes has been documented in more easily discernible paleopolyploids, which are characterized by highly diploidized genomes after several rounds of whole-genome duplications (WGD) at the root of the vertebrate (11, 12). However, research on recurrent polyploids, which are much more difficult to disentangle bioinformatically and experimentally, has seriously lagged behind that on animal paleopolyploids (13).

Viperin (also known as Vig1 in rainbow trout) was first identified as an antiviral protein induced by human cytomegalovirus in 2001 (14, 15). It belongs to the radical S-adenosylmethionine (SAM) enzyme family (14, 16, 17) and is composed of three distinct domains (18). Its N-terminal domain contains an amphipathic α-helix, which localizes Viperin to the ER, lipid droplets, or mitochondria (19–21). The central SAM domain has a characteristic CxxxCxxC motif responsible for binding proteins containing an iron-sulfur cluster (22, 23). The C-terminal domain is highly conserved, but its role remains poorly defined (24). This domain has been proposed to be involved in protein–protein interactions (19).

Viperin is known as an interferon (IFN)-stimulated gene (ISG) with broad-spectrum antiviral activities. Its expression can be induced by lipopolysaccharide (LPS), polyinosinic:polycytidylic acid [poly (I:C)], or various viruses (19, 25). Studies on mammalian Viperin have shown that its antiviral mechanisms appear to be virus-specific, owing to the distinct infection routes and replication strategies of different viruses (26). Recently, Ghosh and Marsh (2020) listed the different viruses reported to be restricted by viperin and the known mechanisms of restriction, and summarized two major ways of the antiviral activities of viperin (27). Viperin has been found to interact with a wide variety of host and viral proteins, and the complex network of interactions inhibits viral RNA transcription and replication (28–30), interrupts viral particle assembly and maturation (14), or impairs viral budding and release (20, 31, 32). Another way relies on the catalytic activity of Viperin to generate 3′-deoxy-3′,4′-didehydro-CTP (ddhCTP), which can directly interfere with RNA synthesis and the replication of flaviviruses (33). Similar to mammal, fish *viperin* is considered an important antiviral gene because its expression is also induced by LPS, poly(I:C), or viruses (15, 34–40). However, little is known about the molecular mechanism underlying the antiviral effect of fish Viperin.

Gibel carp (*Carassius gibelio*), widely distributed across the Eurasian continent, can reproduce by unisexual gynogenesis or sexual reproduction (41–43). Along with a large-scale culture of several improved varieties, such as allogynogenetic gibel carp “CAS III” (clone A^+^) and “CAS III” (clone F) (44, 45), gibel carp has become one of the most important aquaculture species in China with about 3 million tons of annual production capacity (46). However, a serious outbreak of an epizootic disease caused by *Ca*HV has resulted in enormous economic losses since 2012 (47). In previous studies, we identified distinct immune responses and the differential expression of innate and adaptive immune genes among three clones (A^+^, F, and H) after *Ca*HV challenge (48–51). Eight IFN system genes, including *viperin*, were identified as candidate resistant-related genes for disease-resistance breeding of gibel carp. *Viperin* was sharply upregulated among three clones at post infection (52). Additionally, gibel carp has over 150 chromosomes and is considered an auto-allo-hexaploid in comparison with goldfish (*Carassius auratus*) with 100 chromosomes (53, 54). It had been speculated that two rounds (2R) of polyploidy events, an allotetraploidy and an autotriploidy, occurred during its evolution (55). *Viperin* unigenes in transcriptomic data have been classified into two divergent homeologs with biased expression after *Ca*HV infection (52). It is necessary to further analyze expression and function divergence of the two *viperin* homeologs. Therefore, in this study, we first characterized two gibel carp *viperin* homeologs (*Cgviperin-A* and *Cgviperin-B*), and then investigated their divergent antiviral mechanisms through subcellular localization, overexpression, Co-IP, and *in vitro* ubiquitination assay.

## Materials and Methods

### Cells, Gibel Carp, and Virus

Fish cell line *Carassius auratus* L. blastulae embryonic (CAB) cell was cultured at 28°C in 5% CO2 in medium 199 (Invitrogen) supplemented with 10% fetal bovine serum (FBS) (Invitrogen) as described previously (56–58). Female gibel carp clone F (about 67.68 ± 2.16 g) were obtained from the National Aquatic Biological Resource Center (NABRC), Institute of Hydrobiology (IHB), Chinese Academy of Sciences (CAS). *Ca*HV was provided by Prof. Q. Y. Zhang (Institute of Hydrobiology, Chinese Academy of Sciences) and propagated by intraperitoneal injection into healthy gibel carp. The isolation method of *Ca*HV was used as previously described (48, 59). Before sampling, fish were deeply euthanized by overdosed anesthesia styrylpyridine (30–50 mg/L; Aladdin, Shanghai, China) and immediately cut off spinal cord adjacent to the head. All procedures were approved by the Institutional Animal Care and Use Committee of IHB, CAS (protocol number 2016-018).

### Gene Identification and Sequence Analysis

According to the genome sequences of gibel carp clone F (CI01000363_00413629_00414546 and ENSONIP00000026093-D2), specific primers (**Supplementary Table S1**) were designed to amplify *viperin* from gibel carp clone F head kidney cDNA library by 3’ and 5’ rapid amplification of cDNA ends (RACE) (SMARTer^®^ RACE 5’/3’ Kit, Clontech).

The cDNA sequences of six *viperin* transcripts were deposited in GenBank (accession numbers from MZ055409 to MZ055414). The specificity of RACE primer was confirmed by sequence analysis. The sequence assembly was performed by DNAstar software, and the assembly validation was confirmed by full-length sequence amplification and sequence analysis. The validation of open reading frame (ORF), multiple sequence alignments, and neighbor-joining (NJ) phylogeny construction were analyzed by using ORF finder (https://www.ncbi.nlm.nih.gov/orffinder/), ClustalW program, and MEGA 7.0 software, respectively.

### Chromosome Preparation and Fluorescence *In Situ* Hybridization

The bacterial artificial chromosome (BAC) clones containing *Cgviperin-A* and *Cgviperin-B* were screened by Polymerase Chain Reaction (PCR). Then, *Cgviperin-A*-BAC-DNA and *Cgviperin-B*-BAC-DNA were labeled by Biotin-Nick Translation Mix (Roche) and DIG-Nick Translation Mix, respectively. Chromosome preparation and FISH analyses were performed as described previously (55, 60). All metaphase chromosomes were counterstained with 4’, 6-diamidino-2-phenylindole (DAPI).

### RNA Extraction and Quantitative Real-Time PCR

RNA extraction and qPCR were performed as described previously (51, 52). Total RNAs from 10 adult tissues (including intestine, gill, muscle, head-kidney, trunk kidney, liver, spleen, thymus, gonad, and brain) were isolated using SV Total RNA isolation System (Promega) according to the manufacturer’s protocols. Subsequently, the primeScript™ RT reagent Kit with gDNA Eraser (TaKaRa) was used to solve the DNA contamination of total RNA. The quantity of total RNA was detected by Nanodrop 2000 (Thermo Scientific), and the quality was assessed by agarose gel electrophoresis. One µg of total RNAs was used to synthesize first-strand cDNAs in a 20 µl reaction volume following the protocol of GoldScript cDNA synthesis Kit (Invitrogen).

qPCR was performed by using iTaq™ Universal SYBR^®^ Green Supermix (Bio-Rad) according to the manufacturer’s protocol. The reference gene, *eukaryotic translation elongation factor 1 alpha 1, like 1* (*eef1a1l1*) (M value=0.74 < 1.5) was selected as the normalizer for qPCR as described previously (52). The primers (**Supplementary Table S1**) used for qPCR analysis were designed with http://biotools.nubic.northwestern.edu/OligoCalc.html. The specificity of each pair of primers was analyzed by sequencing, and the primer efficiency and R^2^ were assessed by constructing standard curve using the dilutions of cDNA as the template. All samples (n = 3) were analyzed in triplicate. The relative gene expression levels were calculated using the 2^−ΔΔCT^ method. IBM^®^ SPSS^®^ statistics 20 software was used for statistical analysis. A probability (*p*) of <0.05 was considered statistically significant.

### Plasmid Constructs

According to the genome sequences of gibel carp clone F (Wang et al., unpublished data), 5’ flanking sequences and partial sequences of exon 1 of *Cgviperin*-A and *Cgviperin*-B (accession numbers from MZ055415-MZ055420) were also amplified from gibel carp clone F. For luciferase activity assays, these sequences were cloned into Kpn I/Xho I sites of pGL3-Basic luciferase reporter vector (Promega) to construct promoter-driven luciferase vector, respectively. For overexpression, the wild type (WT) *Cgviperin-A* ORF, *Cgviperin-B* ORF, and their corresponding sequences of mutants with a C-terminal hemagglutinin (HA)-tag or a C-terminal Flag-tag were cloned into pcDNA3.1(+) vector, respectively. The *Cgviperin-A* ORF and *Cgviperin-B* ORF were also inserted into pEGFP (enhanced green fluorescent protein)-N3 and pDsRed-N1 vector, respectively. The ORF of *Ca*HV gene ORF46R with a C-terminal HA-tag, Flag-tag, or Myc-tag were cloned into pcDNA3.1(+) vector, respectively. *Ca*IFN-Luc, IFN-stimulated response elements (ISRE)-Luc, and ER-DsRed were constructed previously (61). All constructs were confirmed by sequence analysis.

### Transfection and Luciferase Activity Assays

Luciferase activity assays were performed as described previously (49, 50). CAB cells were seeded in 24-well plates overnight and co-transfected with various plasmid constructs at a ratio of 10:10:1 (promoter-driven luciferase plasmid/expression plasmid/Renilla luciferase plasmid pRL-TK) using FuGENE HD Transfection Reagent (Promega). Empty vector pGL3-Basic luciferase reporter vector (Promega) was used as control. If necessary, the cells were transfected again with poly (deoxyadenylic-deoxythymidylic) acid sodium salt [poly (dA:dT)] (Invivogen) or poly (I:C) (Sigma-Aldrich) (1μg/ml) at 24 h post-transfection. Then, the cells were harvested at 24 h post-transfection and lysed according to the Dual-Luciferase Reporter Assay System (Promega). Luciferase activities were measured by a Junior LB9509 luminometer (Berthold, Pforzheim, Germany) and normalized to the amounts of Renilla luciferase activities. The results were representative of more than three independent experiments, and each was performed in triplicate. The significant differences were calculated by IBM^®^ SPSS^®^ statistics 20 software.

### Co-IP Assay and Western Blotting

Co-IP and western blotting were performed as described previously (62). Briefly, the CAB or 293T cells were seeded into 10 cm^2^ dishes overnight, and transfected with different plasmids (a total of 10 μg) indicated on the Figure using FuGENE HD Transfection Reagent (Promega). At 24 h post-transfection, the cells were lysed by radioimmunoprecipitation (RIPA) lysis buffer with protease inhibitor cocktail (Sigma-Aldrich). After removing cellular debris, the supernatants were incubated with 15 μl anti-Flag or anti-Myc affinity gel (Sigma-Aldrich) overnight at 4°C. Immunoprecipitated proteins were resuspended in 100 μl SDS-PAGE protein loading buffer (Beyotime) after collecting by centrifugation and washing three times with lysis buffer. The whole cell lysates (WCL) or immunoprecipitated proteins were separated by 10–15% SDS-PAGE and then transferred to polyvinylidene fluoride membranes (Bio-Rad). The membranes were incubated with anti-β-actin (Cell Signaling Technology) at 1:3,000, anti-Flag/HA (Sigma-Aldrich) at 1:3,000, or anti-Myc (Santa Cruz Biotechnology) at 1:3,000, and then hybridized with the secondary HRP-conjugated anti-mouse IgG or anti-rabbit IgG (Thermo Scientific) at 1:5,000. Results are captured by using an ImageQuant LAS 4000 system (GE Healthcare) and are representative of three independent experiments.

### *In Vitro* Ubiquitination Assay

Ubiquitination assay was performed as described previously (62, 63). Briefly, CAB cells were transiently co-transfected with different overexpression plasmids (a total of 10 μg) indicated on the Figure and 1 μg HA-Ub or HA-Ub-K63O expression plasmids. At 18 h post-transfection, the cells were treated with 20 mM MG132 (Sigma-Aldrich) for 6 h and then were lysed using a RIPA lysis buffer containing 1% SDS and denatured by heating for 10 min. After denaturing and diluting, the supernatants were immunoprecipitated overnight at 4°C with 30 μl anti-Flag or anti-Myc agarose conjugate (Sigma-Aldrich). The whole cell lysates or immunoprecipitated proteins were analyzed by western blot as above described.

## Results

### Molecular Characterization and Biased Expression Patterns of *Cgviperin* Homeologs

**Figure 1:**
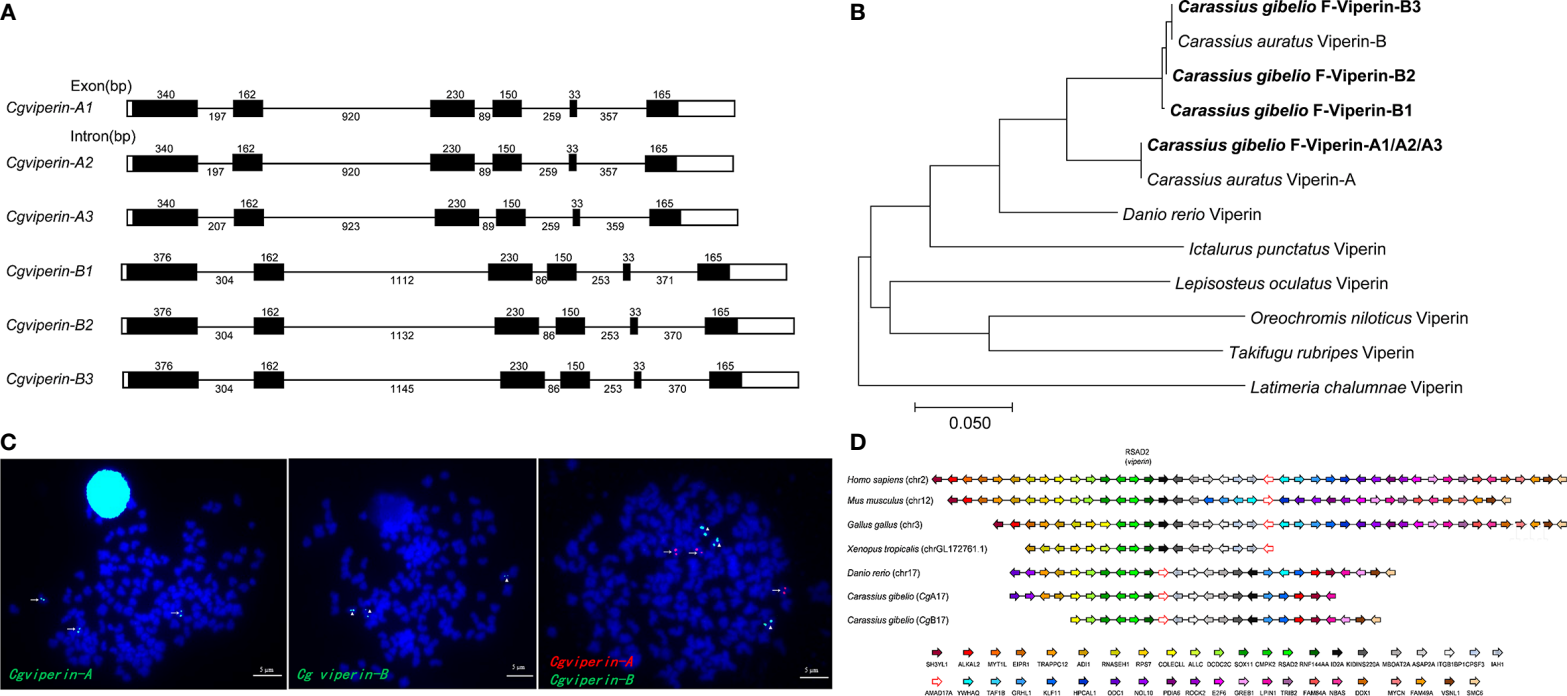
Molecular characterization of *Cgviperin*-A and *Cgviperin*-B in gibel carp. **(A)** Genomic structure of six *Cgviperin* genes. Exons and introns are depicted by rectangle boxes and thick lines, respectively, and ORFs are highlighted by black boxes. Sizes (bp) are indicated upon or below themselves. **(B)** Phylogenetic tree of vertebrate Viperin. **(C)** Localization of *Cgviperin*-A and *Cgviperin*-B on gibel carp metaphase chromosomes. *Cgviperin*-A-BAC-DNA probe was labeled with DIG (green signals) or Biotin (red signals) (indicated by arrows), and *Cgviperin*-B-BAC-DNA probe was labeled with DIG (indicated by arrowheads). All metaphase chromosomes (blue) were counterstained with DAPI. **(D)** Gene synteny of vertebrate *viperin*. Conserved gene blocks are represented in matching colors. Transcription orientations are indicated by arrows.

Six *Cgviperin* transcripts were cloned from the head-kidney of gibel carp clone F using RACE-PCR (**Supplementary Figure S1**). Analysis of multiple nucleotide alignments and their locations in the assembled genome (Wang et al., unpublished data) showed that they were clustered into two homeologs, each possessing three alleles (**Supplementary Figure S1**). The average identities among the three *Cgviperin-A* and *Cgviperin-B* alleles are 99.5 ± 0.3 and 99.4 ± 0.1%, respectively, while the average identity between the *Cgviperin-A* and *Cgviperin-B* homeologs is 91.3 ± 0.1%. The cDNAs of *Cgviperin-A* and *Cgviperin-B* were predicted to encode one *Cg*Viperin-A protein (359aa, *Cg*Viperin-A1/A2/A3) and three *Cg*Viperin-B proteins (371aa, *Cg*Viperin-B1, *Cg*Viperin-B1 and *Cg*Viperin-B3), respectively. Only two amino acid differences were observed among the three *Cg*Viperin-B proteins. *Cg*Viperin-A and *Cg*Viperin-B have about 90.1 ± 0.3% amino acid identity (**Supplementary Figure S2**). Phylogenetic analysis (**Figure 1B**) confirmed that *Cgviperin-A* and *Cgviperin-B* might be a pair of homeologs derived from early allotetraploidy (4R), and they both have three alleles because of late autotriploidy (5R).

Subsequently, the genomic structure and syntenic alignment were analyzed. *Cgviperin-A*s and *Cgviperin-B*s all contain six exons and five introns, and major differences between *Cgviperin-A* and *Cgviperin-B* homeologs exist in the introns (**Figure 1A**). The lengths of five introns vary between the two homeologs, especially the length of first and second introns. The average identity of the corresponding introns among three alleles is 99.3 ± 0.3%, but the average identity between two homeologs is 75.1 ± 0.1%, which is remarkably lower than that of the cDNAs (91.3 ± 0.1%). *Cgviperin-A*s and *Cgviperin-B*s are located on chromosome *Cg*A17 and *Cg*B17 of the gibel carp assembled genome, respectively, and the three alleles of each homeolog in three homologous chromosomes were confirmed by FISH. Three red *Cgviperin-A*s signals and three green *Cgviperin-B*s signals were observed on three different homologous chromosomes when *Cgviperin-A*-BAC-DNA and *Cgviperin-B*-BAC-DNA were simultaneously used as probes (**Figure 1C**). The *Cgviperin* homologous genes were found in zebrafish chromosome (*Danio rerio*) 17 (chr17), human (*Homo sapiens*) chr2, mouse (*Mus musculus*) chr12, chicken (*Gallus gallus*) chr3, and tropical clawed frog (*Xenopus tropicalis*) chrLG172761.1 with a conserved gene cluster (CMPK2-RSAD2/viperin-RNF144AA) (**Figure 1D**). Compared to zebrafish chr17, gibel carp *Cg*A17 and *Cg*B17 both retain most of the zebrafish homologous genes except *ywhaq*.

**Figure 2:**
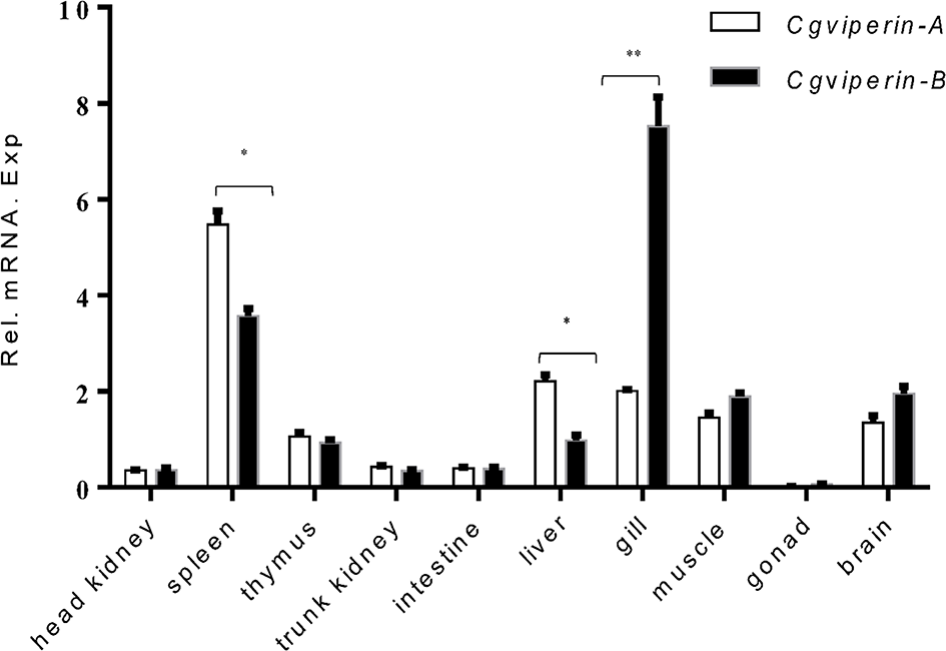
qPCR analysis of *Cgviperin-A* and *Cgviperin-B* expression in adult tissue from healthy gibel carp clone F. *Eukaryotic translation elongation factor 1 alpha 1*, *like 1(eef1a1l1)* was used as control. Each bar represents mean ± standard deviation (SD) (n = 3). *P < 0.05, **P < 0.01.

To confirm the potential antivirus activities of *Cgviperin-A* and *Cgviperin-B*, we compared their expression differences in the adult tissues of healthy individuals (**Figure 2**). *Cgviperin-A* and *Cgviperin-B* were constitutively expressed in the 10 tissues examined. However, obvious expression differences between *Cgviperin-A* and *Cgviperin-B* were observed. Both *Cgviperin-A* and *Cgviperin-B* showed high expression in immune tissue spleen. Additionally, *Cgviperin-A* and *Cgviperin-B* had differentially biased expression among different tissues. *Cgviperin-A* was abundantly expressed and showed higher expression than *Cgviperin-B* in the spleen and liver, while *Cgviperin-B* was highly expressed in the gill.

### *Cg*Viperin-A and *Cg*Viperin-B Both Interact and Colocalize With ORF46R at ER

**Figure 3:**
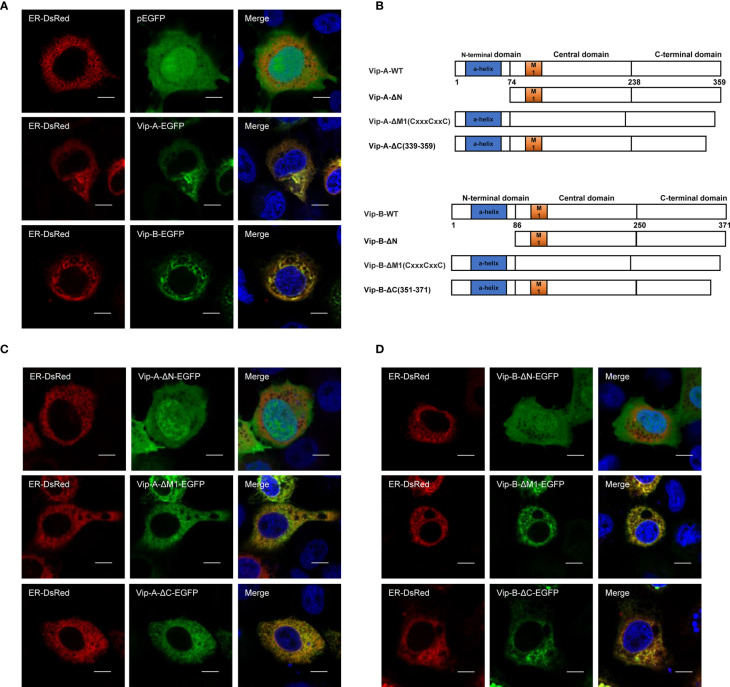
*Cg*Viperin-A and *Cg*Viperin-B colocalize at ER through N-terminus domain. **(A)** Subcellular localization of WT *Cg*Viperin-A, *Cg*Viperin-B, and ER-DsRed. CAB cells were plated onto coverslips in six-well plates and co-transfected *Cg*Viperin-A-EGFP or *Cg*Viperin-B-EGFP with ER-DsRed. After 24 h, the cells were fixed and observed under confocal microscopy. Nuclear was stained with DAPI. Scale bars = 5 μm. **(B)** Diagrammatic representation of WT and three mutants of *Cg*Viperin-A or *Cg*Viperin-B. **(C, D)** Subcellular localization of three mutants of *Cg*Viperin-A **(C)** or *Cg*Viperin-B **(D)** with ER-DsRed.

To further uncover their antiviral mechanisms, we analyzed the roles of different *Cg*Viperin domains and tried to determine their viral interactome. Firstly, we analyzed subcellular the localization of *Cg*Viperin-A and *Cg*Viperin-B in CAB cells (**Figure 3**). After co-transfection of *Cg*Viperin-A- EGFP or *Cg*Viperin-B-EGFP with ER-DsRed, confocal microscopy revealed that both *Cg*Viperin-A and *Cg*Viperin-B (green) mainly overlapped the red ER signals (**Figure 3A**). Then, we performed domain mapping to determine which domain was necessary for *Cg*Viperin-A and *Cg*Viperin-B ER localization. Three mutants of *Cg*Viperin-A and *Cg*Viperin-B were constructed, i.e., Vip-A-ΔN (lacking aa 1-74 in the N-terminal α-helix domain), Vip-A-ΔM (lacking CxxxCxxC motif in the middle SAM domain), and Vip-A-ΔC (339–359) (lacking aa 339-359 in the C terminus) (**Figure 3B**). Similar to WT *Cg*Viperin-A, Vip-A-ΔM and Vip-A-ΔC could localize at the ER, but Vip-A-ΔN was diffusely distributed in the cytoplasm and nucleus (**Figure 3C**). Similar subcellular localization was observed in the mutants of *Cg*Viperin-B (**Figure 3D**). The results indicate that the N-terminal α-helix domain is necessary for the localization of *Cg*Viperin-A and *Cg*Viperin-B at the ER.

**Figure 4:**
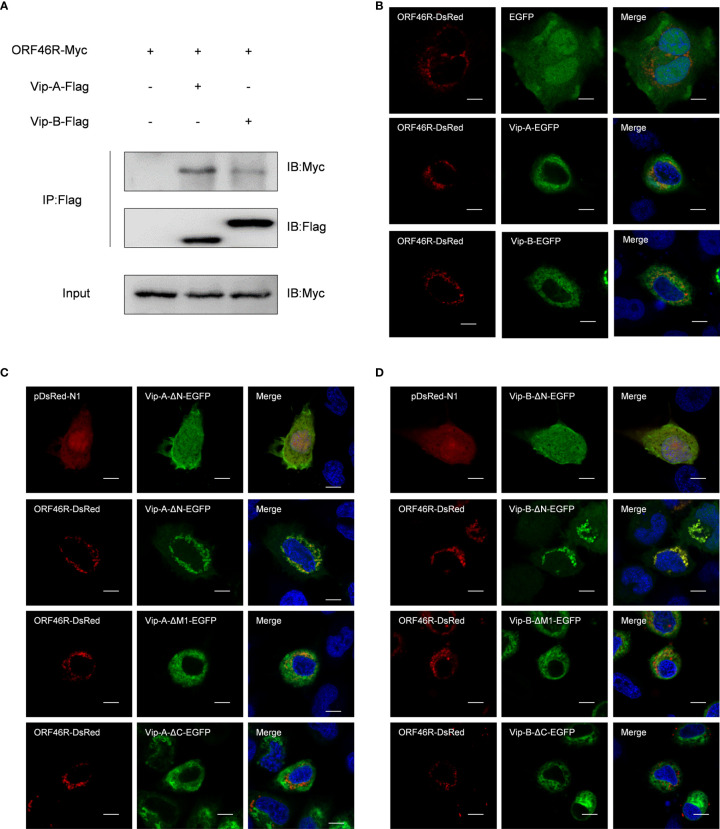
Both *Cg*Viperin-A and *Cg*Viperin-B interact with ORF46R of *Ca*HV. **(A)** Co-IP of Vip-A-Flag or Vip-B-Flag with Myc-ORF46R in CAB cells transfected with the indicated plasmids. Anti-Flag Ab was used for Co-IP. **(B)** Subcellular localization of WT *Cg*Viperin-A, *Cg*Viperin-B, and ORF46R. CAB cells were plated onto coverslips in six-well plates and co-transfected *Cg*Viperin-A-EGFP or *Cg*Viperin-B-EGFP with ORF46R-DsRed. After 24 h, the cells were fixed and observed under confocal microscopy. Nuclear was stained with DAPI. Scale bars = 5 μm. **(C, D)** Subcellular localization of three mutants of *Cg*Viperin-A or *Cg*Viperin-B with ORF46R-DsRed. Scale bars = 5 μm.

Subsequently, to clarify the relevance of *Cg*Viperins and *Ca*HV, the Co-IP experiments were performed, which demonstrated that both *Cg*Viperin-A and *Cg*Viperin-B were efficiently associated with *Ca*HV ORF46R (**Figure 4A**). Next, the subcellular locations of *Cg*Viperins and ORF46R were monitored in CAB cells. Confocal microscopy revealed that the signals of ORF46R were mainly overlapped with those of *Cg*Viperin-A and *Cg*Viperin-B (**Figure 4B**), indicating that both *Cg*Viperin-A and *Cg*Viperin-B partly colocalize with ORF46R at the ER. Finally, we analyzed which domain was essential for the colocalization of *Cg*Viperins with ORF46R. Interestingly, the signals of ORF46R almost overlapped with those of Vip-A-ΔN and partly colocalized with Vip-A-ΔM, but was not with Vip-A-ΔC (**Figure 4C**), and similar subcellular localization was observed among three mutants of *Cg*Viperin-B (**Figure 4D**). These data imply that the C-terminal domain of *Cg*Viperin-A and *Cg*Viperin-B is indispensable for the colocalization with ORF46R.

### *Ca*HV ORF46R Blocks IFN Induction

**Figure 5:**
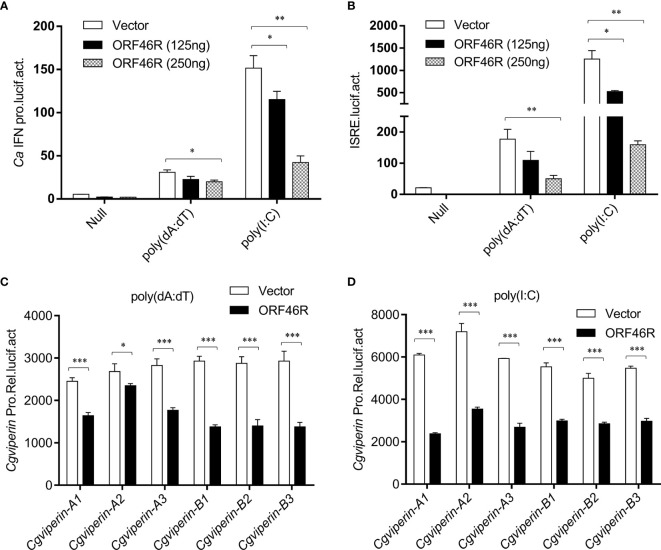
*Ca*HV ORF46R blocks poly (dA:dT) or poly (I:C)-triggered activation of *CaIFN* promoter **(A)**, ISRE luciferase reporter **(B),** and six *Cgviperin* promoters **(C, D)**. CAB cells seeded overnight were co-transfected the empty vector or ORF46R (250 ng) and 25 ng pRL-TK plus 250 ng *Ca*IFNpro-luc or ISRE-luc. At 24 h post-transfection, cells were untreated (null) or transfected with 1 μg poly (dA:dT) or poly (I:C). The luciferase activities were monitored 24 h after stimulation. Each bar represents mean ± standard deviation (SD) (n = 3). *P < 0.05, **P < 0.01, ***P < 0.001.

To obtain a clear picture of the functions of *Cg*Viperin-A and *Cg*Viperin-B in the cellular antiviral immunity, the role of ORF46R for immune response was investigated. As shown in **Figure 5A**, poly(dA:dT) and poly(I:C) could induce the activation of the crucian carp *Ca*IFN promoter (64). However, such induction was significantly impeded by ORF46R in a dose-dependent manner. ISRE, a transcription factor binding motif in the IFN promoter region, facilitates gene transcription (64). Consistently, the same results were also observed in ISRE (**Figure 5B**). For viperin, which was focused in this study, had been identified as an antiviral factor, their promoter activities were checked under ORF46R overexpression as well. The six *Cgviperin* promoters stimulated by poly(dA:dT) or poly(I:C) were blunted ORF46R as expected (**Figures 5C, D**). These results suggest that *Ca*HV ORF46R serves as a negative regulator to block host IFN production or this suppression happened following an induction.

### *Cg*Viperin-A and *Cg*Viperin-B Promote *Ca*HV ORF46R Proteasomal Degradation *via* Decreasing Lysine 63 (K63)-Linked Ubiquitination

**Figure 6:**
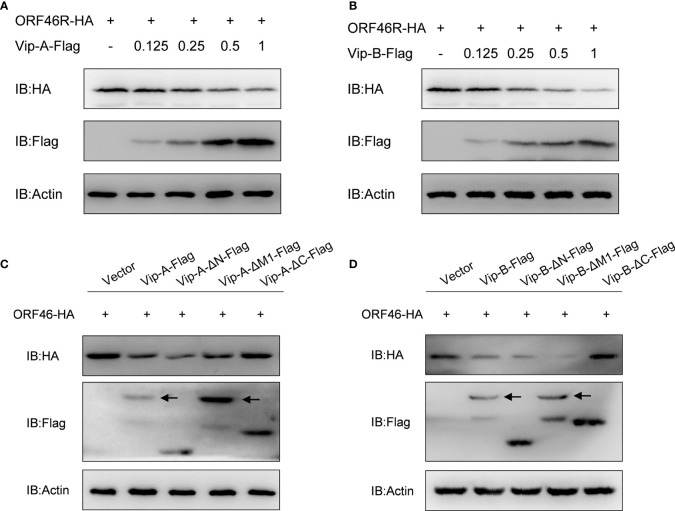
Overexpression of *Cg*Viperin-A and *Cg*Viperin-B induce the reduction of *Ca*HV ORF46R protein *via* its C-terminus. **(A, B)** Overexpression of *Cg*Viperin-A **(A)** or *Cg*Viperin-B **(B)** induced the reduction of ORF46 protein in a dose-dependent manner in CAB cells. CAB cells were transfected with the indicated expression vectors and harvested for western blot analysis after 18–24 h **(C, D)** Domain mapping for *Cg*Viperin-A **(C)** or *Cg*Viperin-B **(D)** domains acting on ORF46R. CAB cells were transfected with the indicated expression vectors (2 μg/well) and harvested for western blot analysis after 18–24 h Diagrammatic representation of three mutants of *Cg*Viperin-A or *Cg*Viperin-B is shown in **Figure 3B**. Data are presented based on three independent experiments.

To determine the effect of *Cg*Viperin-A and *Cg*Viperin-B on the ORF46R of *Ca*HV, Vip-A-Flag or Vip-B-Flag was co-transfected with ORF46R-HA. The overexpression of *Cg*Viperin-A and *Cg*Viperin-B caused a significant reduction of ORF46 protein in a dose-dependent manner, respectively (**Figures 6A, B**). We also used the mutants to determine which domain was indispensable for the capacity of *Cg*Viperin-A or *Cg*Viperin-B to influence the stability of ORF46R. Consistent with WT *Cg*Viperin-A, Vip-A-ΔN and Vip-A-ΔM still could reduce the expression of ORF46R, whereas such an effect was abrogated in the Vip-A-ΔC group (**Figure 6C**). Similar results were obtained in the three mutants of *Cg*Viperin-B (**Figure 6D**), implying that the C-terminus of *Cg*Viperin is necessary for the regulation on ORF46R stability.

**Figure 7:**
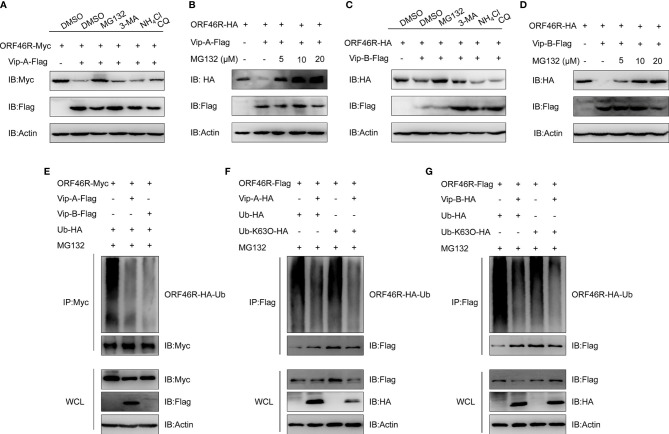
*Cg*Viperin-A and *Cg*Viperin-B promote *Ca*HV ORF46R proteasomal degradation *via* decreasing K63-linked ubiquitination. **(A, C)** Effects of inhibitors on *Cg*Viperin-A **(A)** or *Cg*Viperin-B-mediated **(C)** destabilization of ORF46R. CAB cells were seeded in six-well overnight and transfected with the indicated expression vectors (2 μg/well). The cells were treated with the indicated inhibitors at 18 h post-transfection and harvested for western blot analysis with the anti-Flag and anti-β-actin Abs. **(B, D)** ORF46R degradation induced by *Cg*Viperin-A **(B)** or *Cg*Viperin-B **(D)** was rescued by MG132 in a dose-dependent manner. CAB cells were transfected with the indicated expression vectors (2 μg/well) and treated with DMSO or MG132 (5, 10, or 20 μM) for 6 h at 18 h post-transfection. Then, the WCL were subjected to western blot analysis with the indicated Abs. **(E)** *Cg*Viperin-A and *Cg*Viperin-B decreased the ubiquitination of ORF46R. CAB cells were seeded in 10 cm^2^ dishes and transfected with 5 μg ORF46R-Myc, 4 μg *Cg*Viperin-A-Flag or *Cg*Viperin-B-Flag, and 1 μg HA-Ub and were treated with DMSO or MG132 for 6 h at 18 h post-transfection. After IP with anti-Myc-affinity gels, the immunoprecipitates and WCL were analyzed by western blot analysis with the indicated Abs. **(F, G)** *Cg*Viperin-A **(F)** and *Cg*Viperin-B **(G)** decreased the K63-linked ubiquitination of ORF46R. CAB cells were seeded in 10 cm^2^ dishes were co-transfected with the indicated expression vectors (5 μg ORF46R-Flag, 4 μg *Cg*Viperin-A-HA, or *Cg*Viperin-B-HA) and 1 μg HA-Ub or Ub-K63O-HA, and were treated with MG132 for 6 h at 18 h post-transfection. After IP with anti-Flag-affinity gels, the immunoprecipitates and WCL were analyzed by western blot analysis with the indicated Abs. Data are presented based on three independent experiments.

To determine how *Cg*Viperin-A and *Cg*Viperin-B regulate ORF46R at the protein level, a proteasome inhibitor (MG132), two lysosomal inhibitors [NH_4_Cl and CQ (Chloroquine)], and an autophagosome inhibitor 3-methyladenine (3-MA) were used to examine the manner of *Cg*Viperin-A and *Cg*Viperin-B-mediated ORF46R degradation. In comparison with the control group (DMSO treatment), MG132 could effectively inhibit the degradation of ORF46R induced by *Cg*Viperin-A (**Figure 7A**). Significantly, the reduction of ORF46R was rescued by MG132 dose-dependently (**Figure 7B**). Similar results were also observed in *Cg*Viperin-B group (**Figures 7C, D**). These observations suggest that both *Cg*Viperin-A and *Cg*Viperin-B could promote the proteasomal degradation of ORF46R. Protein ubiquitination is critical for the proteasomal degradation pathway (65). Whether *Cg*Viperin-A and *Cg*Viperin-B induce the polyubiquitination of ORF46R was further investigated. As shown in **Figure 7E**, both *Cg*Viperin-A and *Cg*Viperin-B suppressed the polyubiquitination of ORF46R. Previous studies suggested that target proteins were stabilized by K63-linked polyubiquitin chains (66, 67). Therefore, we tested a ubiquitin mutant, K63-only (K63O) (in which lysine at position 63 was ubiquitinated and linked with polyubiquitin chains, while all other lysine residues were mutated to arginine residues), to determine whether the attenuation of ORF46R was mediated by the decrease of K63-ubiquitination. Consistent with prediction, the K63-linked ubiquitination of ORF46R was significantly impaired under *Cg*Viperin-A regulation (**Figure 7F**). Similar results were also obtained in *Cg*Viperin-B group (**Figure 7G**). The above results suggest that both *Cg*Viperin-A and *Cg*Viperin-B promote ORF46R proteasomal degradation *via* decreasing K63-linked ubiquitination.

### *Cg*Viperin-B Mediates Autophagic Degradation of *Ca*HV ORF46R

**Figure 8:**
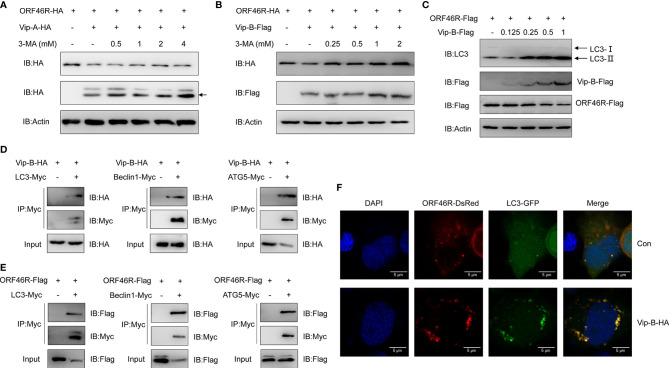
*Cg*Viperin-B mediates degradation of *Ca*HV ORF46R *via* an autophagic pathway. **(A, B)** Effects of 3-MA on *Cg*Viperin-A- **(A)** or *Cg*Viperin-B-mediated **(B)** destabilization of ORF46R. CAB cells were transfected with the indicated expression vectors (2 μg/well) and treated with DMSO or 3-MA (0.5, 1, 2, or 4 mM in A and 0.25, 0.5, 1, or 2 mM in B) for 6 h at 18 h post-transfection. Then, the WCL were subjected to western blot analysis with the indicated Abs. **(C)** Effects of *Cg*Viperin-B on the conversion of LC3B-I to LC3B-II. CAB cells were transfected with increasing amounts of *Cg*Viperin-B-Flag (0.125, 0.25, 0.5, and 1 μg), analyzed by western blot analysis with the indicated Abs. **(D, E)** Co-IP of Vip-B-HA **(D)** or ORF46R-Flag **(E)** with LC3-Myc, Beclin1-Myc, and ATG5-Myc in HEK 293T cells transfected with the indicated plasmids. Anti-Myc Ab was used for Co-IP. **(F)** Subcellular colocalization of ORF46R and *Cg*Viperin-B with LC3-DsRed. CAB cells were plated onto coverslips in six-well plates and co-transfected with the indicated plasmids. After 24 h, the cells were fixed and observed under confocal microscopy. Nuclear was stained with DAPI. Scale bars = 5 μm.

In **Figure 7C**, we noticed that 3-MA could efficiently block ORF46R degradation induced by *Cg*Viperin-B, implying that *Cg*Viperin-B might promote ORF46R degradation through autophagosome pathway, which was distinguished with *Cg*Viperin-A, meaning that the *Cg*Viperin-A and *Cg*Viperin-B functional divergence occurs during the progression of ORF46R degradation. To identify our hypothesis, 3-MA treatment was employed for ORF46R degradation experiments subsequently. Consistent with the results above, increasing doses of 3-MA could not block the ORF46R degradation induced by *Cg*Viperin-A (**Figure 8A**), but *Cg*Viperin-B-mediated ORF46R degradation was obviously rescued by 3-MA in a dose-dependent manner (**Figure 8B**). These results suggest that *Cg*Viperin-B degrade ORF46R *via* an autophagic pathway that is absent in *Cg*Viperin-A. To further illustrate the exact mechanism in this process, we identified that the conversion of Microtubule-associated Protein 1A/1B-Light Chain 3 (LC3)-I to LC3-II was promoted by *Cg*Viperin-B, which means that autophagy is enhanced by the overexpression of *Cg*Viperin-B (**Figure 8C**). Co-IP assays showed that *Cg*Viperin-B interacted with many autophagic components, such as LC3, Beclin1, and autophagy-related gene 5 (ATG5) (**Figure 8D**). Meanwhile, interactions between ORF46R and these autophagic components were also confirmed (**Figure 8E**). Confocal immunofluorescence analysis showed that *Cg*Viperin-B overexpression caused the aggregation of LC3-GFP, demonstrating that cellular autophagy is enhanced (**Figure 8F**). These results suggest that *Cg*Viperin-B mediates the autophagic degradation of ORF46R, which is not possessed by *Cg*Viperin-A.

## Discussion

After WGD, duplicated genes step into complex and dynamic evolutionary routes under relaxed purifying selection. One of the two duplicates may be fractionated (deleted), or both may be retained, or diverge and evolve (sub- or neofunctionalization) (3). Recently, we revealed the biased expression and sub-/neofunctionalization of multiple duplicated *foxl2* homeologs and alleles in gibel carp (60). Innate immunity plays a vital role in the first barrier protecting the host from invading viruses (68). In this study, we focused on *viperin*, which is increasingly considered as a central player in the mammal antiviral response (27), and elucidated the divergent antiviral mechanisms of two homeologs in a polyploid fish.

In addition to the “fish-specific” (3R) WGD event about 320–350 million years ago (Mya), gibel carp was supposed to be experienced two extra rounds of polyploidy [an early allo-polyploidy (4R) and a late auto-polyploidy (5R)] at approximately 18.49 and 0.51 Mya, respectively (55, 60, 69, 70). In this study, we identified two homeologs of *Cgviperin*s with about 91% identity, and each homeolog has three alleles with high identities (≥99.0%) and located on three homologous chromosomes separately (**Supplementary Figure S1** and **Figure 1C**). Compared to zebrafish, our results (**Figures 1B, D**) indicated that the duplication of *Cgviperin*, same as gibel carp other genes (i.e., *dmrt1*, *foxl2*, *nanos2* and *bmp15*) (55, 60, 69, 70), may derive from the early allotetraploidy (4R) and a late autotriploidy (5R) event. In addition, *Cgviperin-A* and *Cgviperin-B* showed a different tissue-dependent constitutive expressions, suggesting A or B homologs might occur dominant or biased expression of homeologs during gibel carp evolution. In our previous study, we also found that *Cgviperin-A* and *Cgviperin-B* exhibited a biased expression pattern after *Ca*HV infection. Significantly, the upregulation folds of them in clone A^+^, F, and H were related to their resistance ability to *Ca*HV, progressively increasing from susceptible clone to resistant clone at 1 dpi (52), which implies that *Cgviperin-A* and *Cgviperin-B* might play an important role in the battle between gibel carp and *Ca*HV.

Consistent with other vertebrate Viperin, both *Cg*Viperin-A and *Cg*Viperin-B contain three domains. The N-terminal domain shows considerable variability between species and aims at the localization of Viperin to the ER, lipid droplets, or mitochondria (19–21). Major sequence differences between *Cg*Viperin-A and *Cg*Viperin-B also exist in their N-terminal region (**Supplementary Figure S2**), and the two N-terminal truncated mutants (Vip-A-ΔN and Vip-B-ΔN) both result in the delocalization of Viperin from the ER membrane (**Figure 3**). The other two domains, the middle SAM and C-terminal domains, are highly conserved across species. The role of the C-terminal domain remains poorly defined (24). Mutating the C-terminal tryptophan residue of human VIPERIN could prevent its binding to iron-sulfur cluster–installing protein CIAO1 (71, 72). In this study, the C-terminal truncated mutants (Vip-A-ΔC and Vip-B-ΔC) failed to colocalize with and degrade *Ca*HV ORF46R (**Figures 4C, D**, **6C, D**), implying that the C-terminus is necessary for *Cg*Viperin-ORF46R interactions.

Mammalian Viperin has been found to interact with three groups of proteins: host proteins involved in innate immune signaling [i.e., Interleukin 1 Receptor Associated Kinase 1 (IRAK1), TNF Receptor Associated Factor 6 (TRAF6), Stimulator of Interferon Genes (STING), and TBK1], host metabolic enzymes [i.e., Farnesyl Pyrophosphate (FPPS) and Microsomal Triglyceride Transfer Protein (MTP)], and viral structural and non-structural proteins [i.e., Non-structural Protein 3 (NS3) and NS5A of flaviviruses] (27). The interactions between mammalian Viperin and viral proteins appear idiosyncratic. Viperin can inhibit viral RNA replication by interfering with the interactions between host proteins and viral non-structural proteins (28–30), inducing the degradation of viral proteins (27, 73), or acting through Fe‐S cluster‐dependent enzymatic activity (72). In this study, we found that both *Cg*Viperin-A and *Cg*Viperin-B could interact and promote *Ca*HV ORF46R protein degradation (**Figures 4A** and **6**). Our preliminary study showed that *Ca*HV ORF46R is a negative regulator of host IFN production because the overexpression of ORF46R could inhibit activation of the *Ca*IFN promoter and ISRE luciferase reporter activity stimulated by poly (dA:dT) (**Figure 5**). Up to now, the domain and exact function of ORF46R are unknown. By bioinformatics analysis, we did not find any known domain in ORF46R and any viral protein similar to ORF46R that had been studied. Further research will be required to determine which region can interact with *Cg*Viperins and to reveal the exact function of ORF46R in *Ca*HV invasion, replication, assembly, or release, especially in *Ca*HV evasion from the host IFN response.

Among the diverse antiviral mechanisms, one emerging theme is that Viperin seems to promote the ubiquitin-dependent proteasomal degradation of some target proteins (27). For example, human VIPERIN can promote the degradation of viral NS5A through the proteasomal degradation pathway (27). It was also found to be capable of stimulating K63-linked polyubiquitination of IRAK1, which phosphorylated interferon regulatory factor (IRF) 7 and ultimately led to IFN production (74). In this study, we found that both *Cg*Viperin-A and *Cg*Viperin-B could promote *Ca*HV ORF46R proteasomal degradation, most likely by decreasing the K63-linked ubiquitination of ORF46R (**Figure 7**). Our findings suggest that *Cg*Viperin-A and *Cg*Viperin-B, like their human homologous genes, may have a general role in engaging with E3 ubiquitin ligases, which are responsible for tagging proteins with polyubiquitin chains. The ubiquitin molecule contains seven lysine residues (K6, K11, K27, K29, K33, K48, and K63) and an amino terminus (M1), which can conjugate with other ubiquitin molecules to form polymeric chains (66, 67). Among them, the K63-based polymeric chain is one of the best described codes and mainly involves in signal transduction, protein sorting and trafficking, and enzymatic activity (67). A growing number of studies suggest that ubiquitin chains play an important role in the modulation of innate immunity (75). Altogether, our results suggest that the overexpression of *Cg*Viperin-A or *Cg*Viperin-B can decrease the K63-linked ubiquitination of endogenous ORF46R protein, which reduces the stability of ORF46R protein and ultimately results in its degradation by the proteasome. There is still much to study on this topic, including which of the ubiquitin ligases *Cg*Viperin and *Cg*Viperin-B interact with and how they cooperate to degrade viral ORF46R.

Eukaryote organisms possess two major cellular degradation machineries: the ubiquitin–proteasome system (UBS) and autophagy-lysosome pathway (ALP) (76, 77). As described above, *Cg*Viperin-A and *Cg*Viperin-B could both promote *Ca*HV ORF46R proteasomal degradation in CAB cells (**Figure 7**). In this study, *Cg*Viperin-B-mediated ORF46R degradation is blocked by autophagy inhibitor 3-MA, and both of them are interacted with the same autophagic components; meanwhile the transformation of LC3 from type I to type II is increased, demonstrating autophagy is one mechanism of *Cg*Viperin-B to degrade viral protein. On the other hand, our data also suggest that ORF46R can also be eliminated by *Cg*Viperin-B during the ubiquitination. Whether there is association between these two mechanisms is still unclear. Which one exerts the major antiviral function is also unknown. Both of that need to be clarified in further study. For unknown reasons, the CAB cells and other cell lines used in our laboratory failed to proliferate *Ca*HV. Therefore, we could not perform the antiviral studies *in vitro* where the proteins are overexpressed or knocked out, followed by a virus infection. In the further study, we will mutate *Cgviperin-A* and *Cgviperin-B*, respectively, in gibel carp by using CRISPR/Cas9 and compare the antiviral differences between them, which would help to complete the story and highlight their antiviral activity.

**Figure 9:**
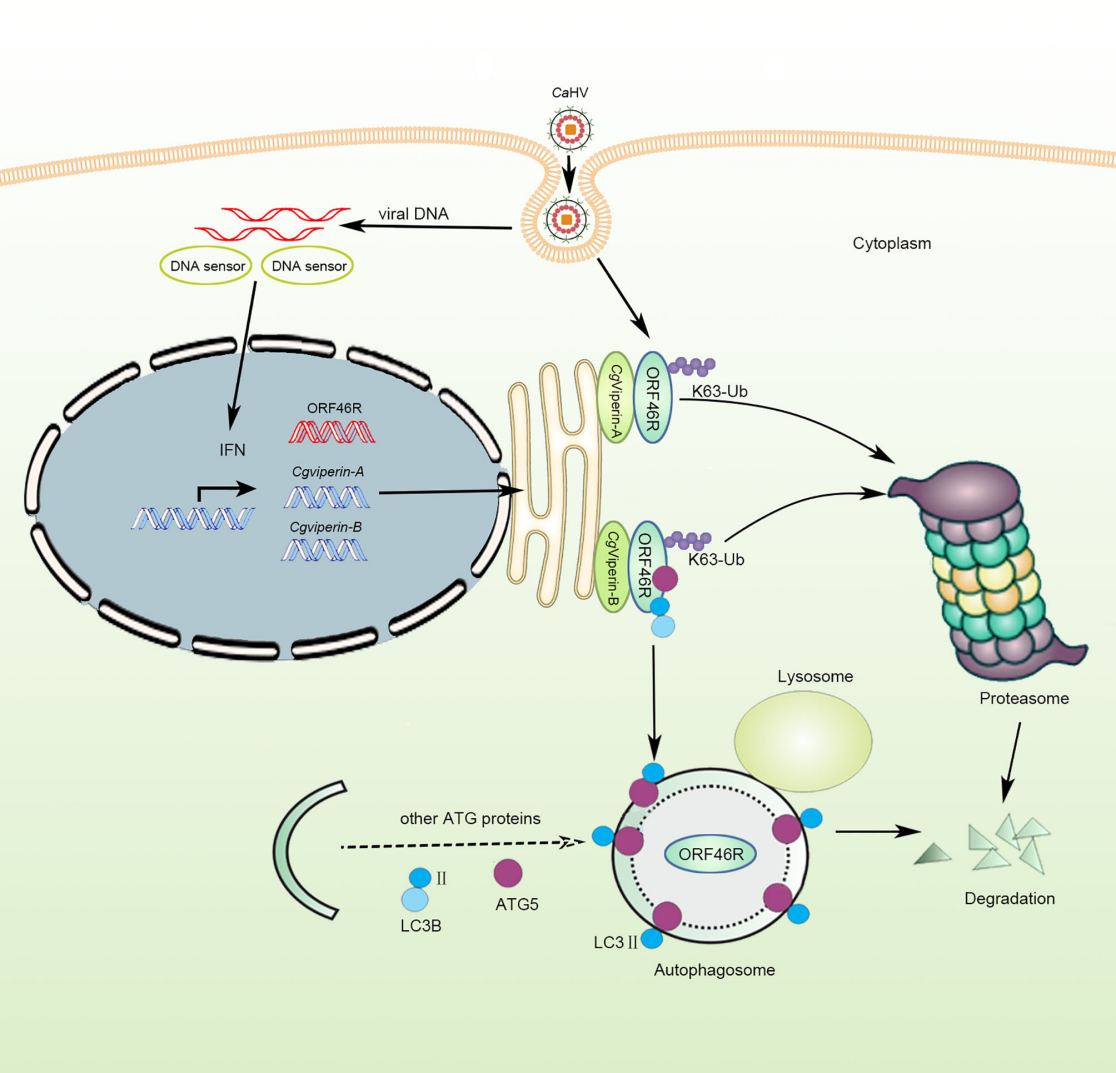
Schematic diagram of divergent antiviral mechanisms of *Cg*Viperin-A and *Cg*Viperin-B in gibel carp resistance against herpesvirus *Ca*HV. Both *Cg*Viperin-A and *Cg*Viperin-B directly interact and promote proteasomal degradation of *Ca*HV ORF46R *via* decreasing K63-linked ubiquitination. In addition, *Cg*Viperin-B induces ORF46R degradation through autophagosome pathway.

Based on the above results of functional analysis *in vitro*, we propose a schematic diagram for divergent antiviral mechanisms of *Cg*Viperin-A and *Cg*Viperin-B in the gibel carp resistance against herpesvirus *Ca*HV (**Figure 9**). After *Ca*HV invasion, both *Cg*Viperin-A and *Cg*Viperin-B could promote the proteasomal degradation of *Ca*HV ORF46R *via* decreasing K63-linked ubiquitination. Additionally, *Cg*Viperin-B also mediated ORF46R degradation through autophagosome pathway. In conclusion, the current findings shed light on the antiviral activities of teleost Viperin as well as the divergent functions and regulative mechanisms of two duplicated *viperin* homeologs. Meanwhile, the above data also imply that the two *viperin* homeologs have subfunctionalized and cooperate to regulate antiviral response.

## Data Availability Statement

The datasets presented in this study can be found in online repositories. The names of the repository/repositories and accession number(s) can be found below: MZ055409 (https://www.ncbi.nlm.nih.gov/nuccore/MZ055409); MZ055410 (https://www.ncbi.nlm.nih.gov/nuccore/MZ055410); MZ055411 (https://www.ncbi.nlm.nih.gov/nuccore/MZ055411); MZ055412 (https://www.ncbi.nlm.nih.gov/nuccore/MZ055412); MZ055413 (https://www.ncbi.nlm.nih.gov/nuccore/MZ055413); MZ055414 (https://www.ncbi.nlm.nih.gov/nuccore/MZ055414); MZ055415 (https://www.ncbi.nlm.nih.gov/nuccore/MZ055415); MZ055416 (https://www.ncbi.nlm.nih.gov/nuccore/MZ055416); MZ055417 (https://www.ncbi.nlm.nih.gov/nuccore/MZ055417); MZ055418 (https://www.ncbi.nlm.nih.gov/nuccore/MZ055418); MZ055419 (https://www.ncbi.nlm.nih.gov/nuccore/MZ055419); MZ055420 (https://www.ncbi.nlm.nih.gov/nuccore/MZ055420).

## Ethics Statement

The animal study was reviewed and approved by the Institutional Animal Care and Use Committee of IHB, CAS (protocol number 2016-018).

## Author Contributions

J-FG, LZ, and C-YM designed the study. C-YM, SL, L-FL, PY, ZL, J-FT, Q-YZ, Z-WW, X-JZ, and G-XW prepared the samples and carried out the experiments. C-YM, LZ, J-FG, SL, and YW analyzed and discussed the results. LZ, J-FG, SL, YW, and C-YM wrote the paper. All authors contributed to the article and approved the submitted version.

## Funding

This work was supported by the Strategic Priority Research Program of the Chinese Academy of Sciences (XDA24030203 and XDA24030104), the National Natural Science Foundation (31772838), and China Agriculture Research System of MOF and MARA (CARS-45-07). The funding bodies had no role in the design of the study and collection, analysis, and interpretation of data and in writing the manuscript.

## Conflict of Interest

The authors declare that the research was conducted in the absence of any commercial or financial relationships that could be construed as a potential conflict of interest.

## Publisher’s Note

All claims expressed in this article are solely those of the authors and do not necessarily represent those of their affiliated organizations, or those of the publisher, the editors and the reviewers. Any product that may be evaluated in this article, or claim that may be made by its manufacturer, is not guaranteed or endorsed by the publisher.

## Supplementary Material

Supplementary Figure S1 Multiple nucleotide sequence alignment of six *Cgviperin* transcripts from gibel carp clone F. ORF is highlighted by red box.

Supplementary Figure S2 Multiple amino acid sequence alignment of *Cg*Viperin proteins from gibel carp clone F.

Supplementary Table S1 Primers used in this study.
