# Supplementary figures and images for: Divergent Antiviral Mechanisms of Two Viperin Homeologs in a Recurrent Polyploid Fish
Source: Front Immunol. 2021 Aug 31;12:702971. doi: 10.3389/fimmu.2021.702971 (PMC8438203; doi:10.3389/fimmu.2021.702971)

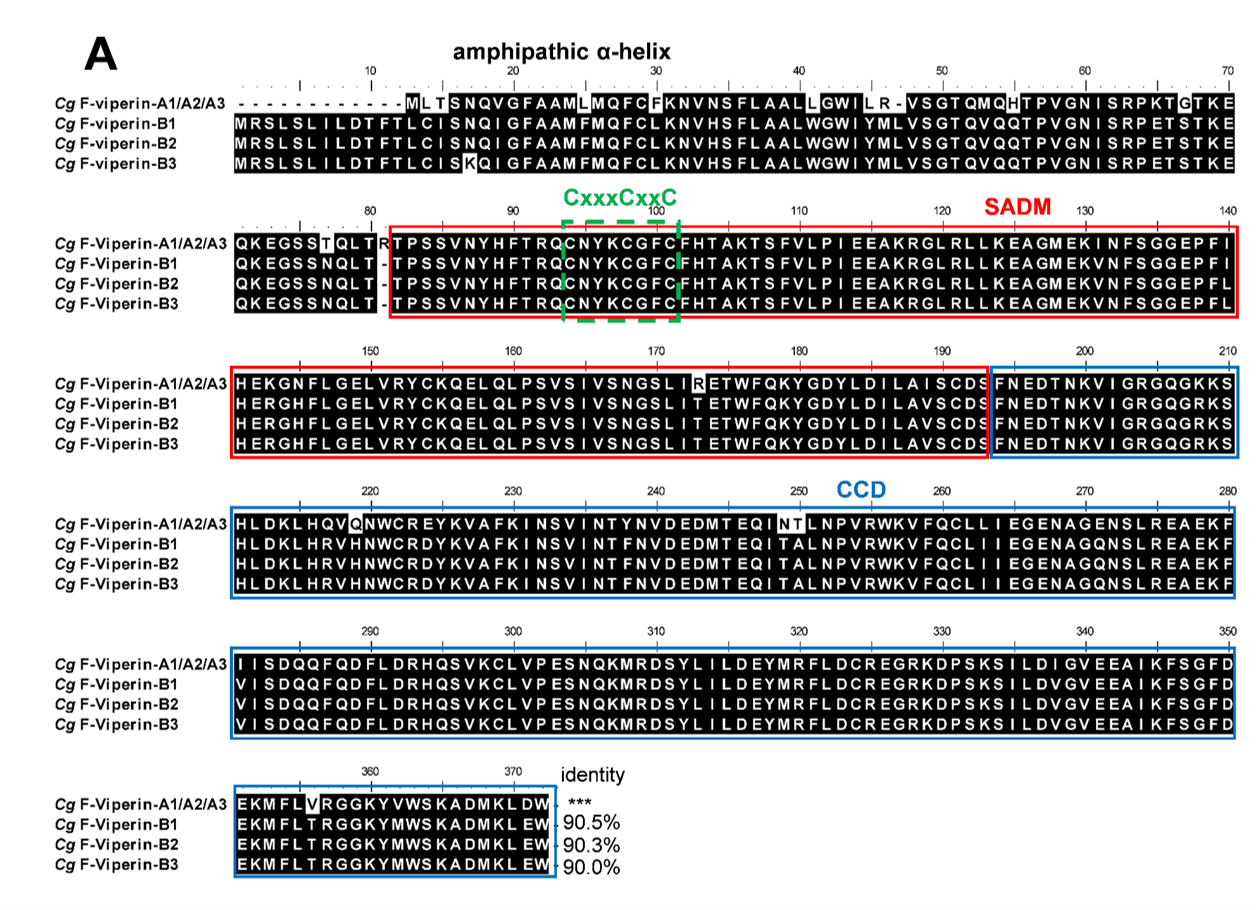

Supplement: Supplementary Figure S2 — Multiple amino acid sequence alignment of CgViperin proteins from gibel carp clone F. [file Image_2.tif]
